# On Carbolic Acid

**Published:** 1864-08

**Authors:** James Watson

**Affiliations:** Physician to the Edinburgh Royal Infirmary


### ON CARBOLIC ACID.

By Dr. James Watson, Physician to the Edinburgh Royal Infirmary.

[Although carbolic acid has been long known it has only been of late used in medicine. We refer our readers to a most able paper by Dr. Crace Calvert, of Manchester, in *Retrospect*, July to Dec. 1863, p. 256.]

It is an external agent—as a disinfectant, antiseptic, and otherwise, that it is most highly spoken of. Mr. Oscar Clayton of London, and Mr. Turner, honorary surgeon to the Manchester Infirmary, have successfully used it as a caustic in the treatment of carbuncle and of foul sloughing sores. Mr. Turner prefers it in the treatment of severe cases of diphtheria to other caustics, as its action does not generally extend below the surface of the parts affected. Mr. Campbell De Morgan, Middlesex Hospital, states that carbolic acid dissolved in glycerine or glacial acetic acid has been found by him very beneficial in the treatment of lupus; and Dr. Whitehead treats lupus successfully with an ointment made of carbolic acid—one half-drachm of acid to one ounce of spermatic ointment. Several respectable surgeons speak favorably of its use as a lotion—one part of the acid to forty parts of water—in all kind of fetid ulcers, gangrenous and offensive sores; and in necrosis it is said to promote the exfoliation of the dead portions of bone. Dr. Calvert states that it is the most powerful preventive of putrefaction with which he is acquainted. He also states that it acts as an anti-ferment, and that he has proved this on an extensive scale—that it prevents the conversion of tannin into gallic acid and sugar. He also tells us that a few drops of this acid added to a pint of fresh urine will preserve it from fermentation or any marked chemical change for several weeks.

Carbolic acid is soluble in any quantity of glycerine, in twice its bulk of glacial acetic acid, in forty times its bulk of water, and forms with a solution of sugar a nice emulsion.

I shall now proceed to record some observations I have made as to its action in the Royal Infirmary.

I have tried carbolic acid as a disinfectant lotion in two cases in the surgical wards. In the first case, the patient, a young girl about thirteen years of age, was suffering from a sloughing ulcer extending from the knee to the ankle. There was a large amount of discharge of the most fetid character. On the application of the carbolic acid as a lotion, one part of the acid to forty of water, the very disagreeable fetor was completely destroyed. Nothing could be more satisfactory than the complete manner in which the disgusting odor was dissipated. The lotion also acted as a stimulant to the part, which to a certain extent took on a healthy action, but in consequence of the amount of discharge exhausting the already weak constitution of the girl, amputation was deemed her only chance of recovery, and this was accordingly performed.

The other instance in which I had carbolic acid applied as a lotion was in the case of a young man who had amputation performed at the middle of the thigh for malignant disease. Very soon after the operation, sloughing set in, accompanied with a fetid discharge. On the application of the lotion, the fetor, as in the first case, was most completely destroyed, the discharge lessened, the sloughs separated, and the parts beneath looked healthy. The lotion continued to be used for several days with the best results as far as its disinfectant and antiseptic qualities were concerned, but unfortunately the patient died, never having thoroughly recovered from the shock of the operation.

I next, along with Dr. Smart, made a number of experiments on urine, to test the antiseptic property of carbolic acid, with the following results:

*First Series of Experiments.*—1st. To ten ounces of diabetic urine I added *no* carbolic acid. After standing for five weeks I found a dense (one inch deep) fungoid mass on the surface of the urine, and the urine having a well-marked odor of decomposition.

2d.—To ten ounces of diabetic urine I added one grain of carbolic acid. At the end of five weeks there was on the surface of the urine a fungoid scum a quarter of an inch deep, but no odor of decomposition.

3d.—To ten ounces of diabetic urine I added three grains of carbolic acid. At the end of five weeks there was no fungoid mass or scum on the urine, which had a perfectly sweet odor.

4th.—To ten ounces of diabetic urine I added five grains of carbolic acid. At the end of five weeks no fungoid mass on surface. Urine sweet, but with a perceptible odor of carbolic acid.

*Second Series of Experiments.*—To five specimens of diabetic urine of ten ounces each, I added the following quantities of carbolic and at the end of three weeks the following were the results:

1st—Of carbolic acid one grain. Urine perfectly free from fungoid mass, and odor perfectly sweet, but there was found a slight precipitate.

2d—Of carbolic acid two grains. Quite free from fungus, but having a deposit, and urine slightly hazy. Free from odor of decomposition.

3d—Of carbolic acid three grains. Deposit, with urine hazy, but quite free from decomposition.

4th. Of carbonic acid four grains. Urine quite translucent, with a deposit less in quantity than in the other quantities of urine, and odor of carbolic acid perceptible.

5th. Of carbolic acid five grains. Urine perfectly translucent and quite free from deposit. Odor of urine sweet, with that of the carbolic acid distinct.

These two series of experiments conclusively show, that carbolic acid is a decided antiseptic, that a very small quantity prevents for several weeks the odor of decomposition—that a slightly increased quantity diminishes the amount of deposit, and a very little more entirely prevents any recognizable change in the composition of the urine from taking place.

I shall next draw attention to a case of favus treated with carbolic acid in the Clinical Wards.

Peter Russell, aged fourteen. On admission, head was found completely covered with favus crust, and on microscopic examination the parasite—*achorion Schonleini*—peculiar to this disease was discovered. The boy's head was shaved and then poulticed for two days. A solution of carbolic acid in glycerine (in the proportion of one part of the acid to twenty-five parts of glycerine) was then ordered to be applied to his head, morning and evening, and to be continued daily.

Before the application of this lotion the entire scalp was of a crimson color, but under this treatment the color of the scalp became gradually fainter, and at the end of five weeks distinct tracts of scalp quite free from redness were found, and the remaining parts were much paler in color. The hair is now growing vigorously, and over the whole surface of the scalp not a trace of the favus crust can be detected.

The boy's general health is much improved since coming into the hospital.

So satisfactory was the result obtained in the last reported case considered, that the same treatment is now being pursued in another case of favus, with this difference, that in this instance the lotion is stronger, being composed of one part of acid to fifteen of glycerine.

The young man, Peter Ward, aged seventeen, has had the disease for nine years. On admission his head was covered with favus crust. The head having been shaved and poulticed, the above lotion was applied as in the former case, morning and evening. After being treated in this way for fourteen days the scalp was seen to be much less red and the hair growing vigorously. At the roots of the hair there are still small circumscribed patches of crimson scalp, but no trace of crust is to be found over the entire head.—*Edinburgh Med. Journal, Jan.* 1864, *p.* 625—*Braithwaite's Retrospect.*

